# Editorial: Porcine respiratory disease complex: dynamics of polymicrobial infections, synergistic effects and management strategies

**DOI:** 10.3389/fvets.2023.1329073

**Published:** 2023-11-29

**Authors:** Natalia Ramos, Marina Sibila, Victor Neira

**Affiliations:** ^1^Sección Virología, Instituto de Biología, Facultad de Ciencias, Universidad de la República, Montevideo, Uruguay; ^2^IRTA, Centre de Recerca en Sanitat Animal (CReSA, IRTA-UAB), Campus de la UAB, Cerdanyola del Vallès, Spain; ^3^Unitat Mixta d'Investigació IRTA-UAB en Sanitat Animal, Centre de Recerca en Sanitat Animal (CReSA), Campus de la Universitat Autònoma de Barcelona (UAB), Cerdanyola del Vallès, Spain; ^4^WOAH Collaborating Centre for the Research and Control of Emerging and Re-Emerging Swine Diseases in Europe (IRTA-CReSA), Bellaterra, Barcelona, Spain; ^5^Departamento de Medicina Preventiva Animal, Facultad de Ciencias Veterinarias y Pecuarias, Universidad de Chile, Santiago, Chile

**Keywords:** porcine respiratory disease complex, polymicrobial infections, infection interaction dynamics, patterns of co-infection and superinfection, management strategies

Porcine respiratory disease complex (PRDC) is a major cause of production losses in the global swine industry. The disease is often multifactorial and polymicrobial, resulting from infection with different combinations of pathogens (including viruses, bacteria, and parasites), in addition to non-infectious factors such as environmental conditions, animal age, population size, management strategies and genetics. Given the complex nature of PRDC and the lack of comprehensive research on the subject, the objectives of this Research Topic were to provide insights into pathogen detection in swine respiratory disease, to identify patterns of co-infection, superinfection and opportunistic pathogens, and to establish monitoring methods and preventive measures.

The first study that contributed to the aim of this Research Topic focused on the identification of several pathogens in swine with typical clinical respiratory signs from farms in China, one of the leading pork producing countries (Sun et al.). The authors analyzed 1,307 samples from 269 farms in 17 provinces, screening for the most important viral and bacterial pathogens. Viruses such as porcine reproductive and respiratory syndrome virus (PRRSV), porcine circovirus type 2 (PCV-2), and pseudorabies virus (PRV) were detected in tissue samples (lungs, inguinal lymph nodes and tonsils) by PCR. In addition, five common respiratory bacteria [*Streptococcus suis, Glaesserella (Haemophilus) parasuis, Pasteurella multocida, Actinobacillus pleuropneumoniae, and Bordetella bronchiseptica*] were isolated and identified from nasal swabs, trachea, lung, spleen, brain, and joint fluid samples. The results indicated that PRRSV, PCV2, *S. suis* and *G. parasuis* were the most commonly detected pathogens in pigs with PRDC in China. Furthermore, the findings unveiled that co-infections of two or more PRDC-associated pathogens with strong positive correlations were common in China. These included double infections like PRRSV and *G. parasuis*, PRRSV and *P. multocida*, PCV-2 and *P. multocida*, and also triple infections such as PRRSV-PCV-2 and *G. parasuis* and PRRSV-PCV-2 and *P. multocida*. This study also highlighted the identification of certain pathogens in relation to the age of the animals; for example, PRRSV, PCV2, and *G. parasuis* exhibited the highest detection rates in nursery pigs, while *P. multocida* and *A. pleuropneumoniae* had the highest detection rates in fattening pigs.

Another article published in the present topic was centered on the identification of viral pathogens in nasal swab from 55 cases of respiratory disease outbreaks under the suspicion of swine influenza A virus (swIAV) in nurseries from Spain and Portugal (Martín-Valls et al.). Notably, the authors conducted screenings for a wide array of respiratory viruses, including: swIAV, PCV-2, PRRSV, influenza B (IBV) and D (IDV), swine orthopneumovirus (SOV), porcine parainfluenza 1 virus (PPIV1), porcine circovirus 3 and 4 (PCV-3, PCV-4), porcine respiratory coronavirus (PRCV), and porcine cytomegalovirus (PCMV). The results indicated that PCV-3, PRRSV and PCMV were the most commonly detected viruses at the herd level (in more than 70% of cases analyzed). However, IBV, IDV, PCV-4, and PPIV1 were not detected in any case. The study delved deeper into the interaction between different viral pathogens, identifying thirty-one combination patterns, which suggested a complex etiology of respiratory disease in pigs. Furthermore, the authors demonstrated that the frequency of a specific pathogen detected in swIAV-positive cases at herd level differed from that in swIAV-negative cases. Interestingly, PRCV, SOV and PCMV were more likely to be found in swIAV-positive herds. On the other hand, PRRSV exhibited a negative correlation with swIAV and PRCV, but a positive correlation with PCMV. Another noteworthy finding from this work was the identification of the circulation of PRCV, PCMV, SOV and PCV-3 initiated in the 1^st^ weeks of life, indicating a significant role for sows.

The third article of this Research Topic was focused on the detection of PRRSV, one of the most commonly detected viruses in respiratory disease (Osemeke et al.). In this work, the use of oral swabs for PRRSV detection by RT-rtPCR was analyzed and compared it to serum samples from weaning-age pig litters. The results revealed that while oral swabs represent easy-to-collect and non-invasive individual sample, they are less sensitive alternative for PRRSV surveillance when compared to the serum samples, which serve as reference.

In the context of PRDC control, this Research Topic also included an article on the comparative evaluation of two bivalent vaccines containing PCV-2 and *Mycoplasma hyopneumoniae* (Cho et al.), a widely used strategy for preventing clinical respiratory signs on pig farms. One vaccine contained PCV-2a and the other contained PCV-2b. The results obtained showed that both bivalent vaccines were viable options for controlling subclinical PCV-2d infection and enzootic pneumonia swine farms. Additionally, both vaccines induced protective immunity by reducing PCV2 blood viral load and *M. hyopneumoniae* laryngeal load, while mitigating pulmonary and lymphoid lesions.

Finally, a review of the dynamics of polymicrobial infections in PRDCs and related management strategies was a valuable contribution to this Research Topic (Assavacheep and Thanawongnuwech). The authors emphasized that new management strategies have been proposed following the introduction of African swine fever (ASF), particularly in Asian countries, and some of these may inadvertently lead to the re-emergence of existing endemic PRDC pathogens, like PRRSV and PCV-2. However, the strict biosecurity measures required during the ASF era, such as minimizing pig contact through the use of automated feeding systems, artificial intelligence and robotic farming, may also help to eliminate PRDC from farms.

These studies collectively illustrate the diverse array of pathogens identifiable in swine respiratory disease and the intricate dynamics of their interactions. More comprehensive studies monitoring a wide range of pathogens, both viral and bacterial, are increasingly needed to gain a better understanding of PRDC. In addition, consistency in sample selection and analysis of different age groups would be essential to allow more accurate comparisons between studies. Definitely, more research in this area will allow better prevention and control strategies to be developed.

## Author contributions

NR: Writing—original draft, Writing—review & editing. MS: Writing—review & editing. VN: Writing—review & editing.

